# Rates of Neuropsychiatric Disorders and Gestational Age at Birth in a Danish Population

**DOI:** 10.1001/jamanetworkopen.2021.14913

**Published:** 2021-06-29

**Authors:** Yuntian Xia, Jingyuan Xiao, Yongfu Yu, Wan-Ling Tseng, Eli Lebowitz, Andrew Thomas DeWan, Lars Henning Pedersen, Jørn Olsen, Jiong Li, Zeyan Liew

**Affiliations:** 1 Yale Center for Perinatal, Pediatric, and Environmental Epidemiology, Yale School of Public Health, New Haven, Connecticut; 2 Department of Environmental Health Sciences, Yale School of Public Health, New Haven, Connecticut; 3 Department of Clinical Epidemiology, Aarhus University Hospital, Aarhus, Denmark; 4 Department of Biostatistics, School of Public Health, The Key Laboratory of Public Health Safety of the Ministry of Education, Fudan University, Shanghai, China; 5 Yale Child Study Center, Yale School of Medicine, New Haven, Connecticut; 6 Department of Chronic Disease Epidemiology, Yale School of Public Health, New Haven, Connecticut; 7 Department of Clinical Medicine, Aarhus University, Aarhus, Denmark; 8 Department of Biomedicine, Aarhus University, Aarhus, Denmark; 9 Department of Obstetrics & Gynecology, Aarhus University Hospital, Aarhus, Denmark

## Abstract

**Importance:**

Nonoptimal gestational durations could be associated with neurodevelopmental disabilities, yet evidence regarding finer classification of gestational age and rates of multiple major neuropsychiatric disorders beyond childhood is limited.

**Objective:**

To comprehensively evaluate associations between 6 gestational age groups and rates of 9 major types and 8 subtypes of childhood and adult-onset neuropsychiatric disorders.

**Design, Setting, and Participants:**

This cohort study evaluated data from a nationwide register of singleton births in Denmark from January 1, 1978, to December 31, 2016. Data analyses were conducted from October 1, 2019, through November 15, 2020.

**Exposures:**

Gestational age subgroups were classified according to data from the Danish Medical Birth Register: very preterm (20-31 completed weeks), moderately preterm (32-33 completed weeks), late preterm (34-36 completed weeks), early term (37-38 completed weeks), term (39-40 completed weeks, reference), and late or postterm (41-45 completed weeks).

**Main Outcomes and Measures:**

Neuropsychiatric diagnostic records (*International Statistical Classification of Diseases and Related Health Problems, Tenth Revision* codes F00-F99) were ascertained from the Danish Psychiatric Central Register up to August 10, 2017. Poisson regression was used to estimate the incidence rate ratio (IRR) and 95% CI for neuropsychiatric disorders, adjusting for selected sociodemographic factors.

**Results:**

Of all 2 327 639 singleton births studied (1 194 925 male newborns [51.3%]), 22 647 (1.0%) were born very preterm, 19 801 (0.9%) were born moderately preterm, 99 488 (4.3%) were born late preterm, 388 416 (16.7%) were born early term, 1 198 605 (51.5%) were born at term, and 598 682 (25.7%) were born late or postterm. A gradient of decreasing IRRs was found from very preterm to late preterm for having any or each of the 9 neuropsychiatric disorders (eg, very preterm: IRR, 1.49 [95% CI, 1.43-1.55]; moderately preterm: IRR, 1.23 [95% CI, 1.18-1.28]; late preterm: IRR, 1.17 [95% CI, 1.14-1.19] for any disorders) compared with term births. Individuals born early term had 7% higher rates (IRR, 1.07 [95% CI, 1.06-1.08]) for any neuropsychiatric diagnosis and a 31% higher rate for intellectual disability (IRR, 1.31 [95% CI, 1.25-1.37]) compared with those born at term. The late or postterm group had lower IRRs for most disorders, except pervasive developmental disorders, for which the rate was higher for postterm births compared with term births (IRR, 1.06 [95% CI, 1.03-1.09]).

**Conclusions and Relevance:**

Higher incidences of all major neuropsychiatric disorders were observed across the spectrum of preterm births. Early term and late or postterm births might not share a homogeneous low risk with individuals born at term. These findings suggest that interventions that address perinatal factors associated with nonoptimal gestation might reduce long-term neuropsychiatric risks in the population.

## Key Points

**Question:**

Are there associations between gestational age, analyzed in 6 subgroups covering the full range of gestational duration, and the rate of neuropsychiatric diagnoses?

**Findings:**

In this Danish, nationwide, registry-based cohort study, shortened gestational duration was associated with the rate of both child-onset and adult-onset neuropsychiatric diseases. Beyond the traditional threshold of fetal maturity (≥37 weeks), the early term group (37-38 weeks) had a slightly elevated rate of multiple neuropsychiatric disorders compared with the full-term group, whereas the late-term and postterm groups had the lowest rates for most disorders except pervasive developmental disorders.

**Meaning:**

These findings suggest that neuropsychiatric disorders might be associated with factors related to early development and that interventions focusing on perinatal risk factors and obstetric practices might lower the risk for neuropsychiatric disorders in the population.

## Teaser

This cohort study evaluates associations between 6 gestational age groups and rates of 9 major types and 8 subtypes of childhood-onset and adult-onset neuropsychiatric disorders using data from a Danish nationwide registry.

## Introduction

Gestation is a critical period for brain growth and development.^1,2^ Nonoptimal gestational duration might have lifelong health consequences, including neurodevelopmental impairments and psychiatric morbidities.^1,3^ Most epidemiological studies^4,5,6,7,8^ that have found an association between gestational age and neuropsychiatric outcomes have quantified risks according to dichotomous classification of gestational measures using the 37-week cutoff as a marker of maturity. However, the associations between the whole range of gestational age, considering the possible heterogeneity within gestational age groups before and after the cutoff, and multiple neuropsychiatric diseases in childhood and adulthood are less studied. Moreover, although it is recognized that children born very preterm (<32 weeks)^9,10,11^ and preterm (<37 weeks)^12^ are especially vulnerable to neuropsychiatric diseases, less is known about potential risk patterns among the term births. For instance, children born within 37 to 41 weeks of gestation have traditionally been considered as a homogeneous, low-risk group,^13^ but emerging evidence has indicated that children born at early term (37-38 weeks) had poorer cognitive outcomes,^14^ lower educational achievement,^15^ and higher rates of mortality^16^ compared with those born at 39 through 41 weeks of gestation. Finally, some associations between postterm pregnancy (>42 weeks) and neurobehavioral^17^ or mental health disorders^18^ have been suggested, yet evidence remains inconclusive.

Using data from Danish national registers, we aimed to evaluate the associations between 6 gestational age groups, considering the full range of gestational duration, from very preterm to postterm birth, and rates of 9 major types and 8 subtypes of childhood-onset and adult-onset neuropsychiatric disorders. We also investigated whether comorbidity of multiple neuropsychiatric disorders was associated with gestational ages at birth.

## Methods

This cohort study was approved by the Danish Data Protection Agency and the institutional review board at Yale University. Informed consent was not required according to Danish law governing registry-based research studies with no participant contact. The Strengthening the Reporting of Observational Studies in Epidemiology (STROBE) reporting guideline was followed.

### Study Population

This population-based, record linkage study included all live-born singletons registered in the Danish Medical Birth Registry (DMBR),^19^ an electronic data register established in 1973 that contains information on maternal and neonatal outcomes for more than 98% of deliveries in Demark.^20^ The unique 10-digit personal identifier assigned to all Danish residents allows linkage between the DMBR and other national registries, including the Danish Psychiatric Central Research Register (DPCR), which has registered electronic information on psychiatric hospital admissions in Denmark since 1969 and outpatient psychiatric visits since 1995.^21^ Singleton births born between January 1, 1978, and December 31, 2016, and who resided in Denmark during the study follow-up from January 1, 1994, to August 10, 2017, were eligible for inclusion. Singleton births with an unlikely short (<20 completed weeks), long (>45 completed weeks), or missing age of gestation were excluded (84 663 births), yielding a total of 2 327 639 individuals aged up to 40 years for analysis.

### Gestational Age

Gestational age recorded in the DMBR in most cases was estimated on the basis of ultrasound examinations done before 24 weeks of gestation conducted by sonographers or the first day of the last menstrual period. Individuals were classified into 6 subgroups according to their gestational age estimates: very preterm (20-31 completed weeks), moderately preterm (32-33 completed weeks), late preterm (34-36 completed weeks), early term (37-38 completed weeks), term (39-40 completed weeks), and late or postterm (41-45 completed weeks).

### Neuropsychiatric Disorders in Offspring

We used *International Statistical Classification of Diseases and Related Health Problems, Tenth Revision *(*ICD-10*) codes^22^ to ascertain neuropsychiatric diagnosis of interest records up to August 10, 2017. We did not include *International Statistical Classification of Diseases and Related Health Problems, Eighth Revision *(*ICD-8*) diagnoses before 1994 to avoid differences in disease classification and missing records of outpatient visits. Considering the duration of follow-up and disease prevalence in the study population, we focused on 9 major types and 8 subtypes of neuropsychiatric diagnoses recorded in the DPCR.^23^ The major neuropsychiatric diseases disorders included (1) mental and behavioral disorders due to psychoactive substance abuse (*ICD-10* codes F10-F19); (2) schizophrenia and related disorders (*ICD-10* codes F20-F29); (3) mood disorders (*ICD-10* codes F30-F39); (4) neurotic, stress-related, and somatoform disorders (*ICD-10* codes F40-F48); (5) eating disorders (*ICD-10* code F50); (6) specific personality disorders (*ICD-10* code F60); (7) intellectual disability (*ICD-10* codes F70-F79); (8) pervasive developmental disorders (*ICD-10* code F84); and (9) behavioral and emotional disorders with onset usually occurring in childhood and adolescence (*ICD-10* codes F90-F98). Among these major groups, we also investigated 8 subtypes of common neuropsychiatric diseases, including (1) mental and behavioral disorders due to alcohol use (*ICD-10* code F10), (2) mental and behavioral disorders due to cannabis use (*ICD-10* code F19), (3) schizophrenia (*ICD-10* code F20), (4) schizoaffective disorders (*ICD-10* code F25), (5) bipolar disorder (*ICD-10* codes F30 and F31), (6) anorexia nervosa (*ICD-10* code F50.0), (7) childhood autism (*ICD-10* code F84.0), and (8) hyperkinetic disorder (*ICD-10* code F90). For each neuropsychiatric disorder, the date of onset was defined as the first day of the initial contact with inpatient or outpatient psychiatric services for the diagnosis of interest.

### Covariates

Factors of importance associated with gestational age and the outcome were selected a priori as covariates. Covariate data were obtained and integrated from multiple Danish national registers. Sex and calendar year of birth (1978-1982, 1983-1987, 1988-1992, 1993-1997, 1998-2002, 2003-2007, 2008-2012, and 2013-2016) were obtained from the DMBR, and maternal age at delivery (12-19, 20-24, 25-29, 30-34, 35-39, and ≥40 years) and maternal country of origin (Denmark or others) were obtained from the Danish Civil Registration Service.^24^ Maternal education level (primary and lower secondary, upper secondary education and academy profession degree, and bachelor’s degree and above) was obtained from the Integrated Database for Labor Market Research.^25^ Parental history of neuropsychiatric disorders (yes or no) was defined as either the mother or the father ever receiving a diagnosis of any neuropsychiatric psychiatric illness (*ICD-10* codes F00-99) recorded in the DPCR before the birth of the index child.

### Statistical Analysis

We used log-linear Poisson regression models to estimate the crude and adjusted incidence rate ratios (IRRs) and 95% CIs for having received a diagnosis of any major or subtypes of neuropsychiatric disorders by the 6 gestational age groups. Term births (39-40 weeks) were used as the reference. We controlled for several covariates, including sex, calendar year of birth, maternal education level, maternal country of origin, maternal age at delivery, and parental history of mental illness in the adjusted models. Because we assessed *ICD-10* diagnoses used since 1994,^26^ person-time follow-up for cohort members started from January 1, 1994, if they were born before 1994, to avoid immortal time bias for missing outcomes before this date.^27^ For those born during or after 1994, the follow-up began at the date of birth. All members were followed until a diagnosis of any neuropsychiatric disorder of interest, death, emigration from Denmark, or the end of follow-up, whichever came first. The median (interquartile range) length of study follow-up for the cohort was 19.0 (15.2-22.5) years. When studying specific types of neuropsychiatric disorders, each disorder was analyzed as an independent outcome. The cohort member was censored when they received the first primary or secondary diagnosis of a given disorder but continued to contribute time at risk for other disorders for which the individual had not received a diagnosis. In sensitivity analysis, we excluded individuals born before January 1, 1995, and re-examined our findings. This was because we did not consider diagnoses not based on the *ICD-10* and because the use of *ICD-10* codes in 1994 might be partial in that transition year. We used multinomial regression model to estimate the odds ratio (OR) and 95% CI for receiving none (reference), only 1, 2 to 3, and 4 or more types of major neuropsychiatric diagnoses according to gestational age groups. Potential sex differences were also evaluated in stratified analysis, and heterogeneity test assessing the 2-sided *P* value of sex and gestational age group product term in multiplicative scale was conducted. We quantified the associations (eg, direction, magnitude, and precision) including *P* value when needed (eg, for heterogeneity). Findings are based on assessing the effect estimates and the overall patterns. Only individuals with complete covariate data were included, given the small number of missing values (<3% of individuals). All analyses were performed using SAS statistical software version 9.4 (SAS Institute). Statistical analyses were conducted from October 1, 2019, through November 15, 2020.

## Results

Of all 2 327 639 singleton births included (1 194 925 male newborns [51.3%]), 22 647 (1.0%) were born very preterm (20-31 weeks), 19 801 (0.9%) were born moderately preterm (32-33 weeks), 99 488 (4.3%) were born late preterm (34-36 weeks), 388 416 (16.7%) were born early term (37-38 weeks), 1 198 605 (51.5%) were born at term (39-40 weeks, reference), and 598 682 (25.7%) were born late or postterm (41-45 weeks) (Table 1). The incidence rate (per million person-years) and the median age of first diagnosis for the 9 major types and 8 subtypes of neuropsychiatric disorders are in Table 2. The median (interquartile range) age at the time of any neuropsychiatric diagnosis was 17.0 (11.7-22.1) years, with an incidence rate of 5483.3 per million person-years.

**Table 1.** Characteristics of the Study Participants by Gestational Age Subgroups

Category	Newborns, No. (%)					
	Very preterm (20-31 wk)	Moderate preterm (32-33 wk)	Late preterm (34-36 wk)	Early term (37-38 wk)	Term (39-40 wk)	Postterm (41-45 wk)
Birth year						
1978-1982	2068 (9.13)	1958 (9.89)	9854 (9.90)	33 454 (8.61)	162 733 (13.58)	71 535 (11.95)
1983-1987	1733 (7.65)	1557 (7.86)	8031 (8.07)	28 542 (7.35)	118 238 (9.86)	57 967 (9.68)
1988-1992	2708 (7.65)	2363 (7.86)	12 160 (8.07)	45 483 (7.35)	166 629 (9.86)	85 051 (9.68)
1993-1997	3141 (13.87)	2637 (13.32)	14 005 (14.08)	51 729 (13.32)	172 754 (14.41)	90 620 (15.14)
1998-2002	3503 (15.47)	3193 (16.13)	15 278 (15.36)	57 490 (14.80)	157 882 (13.17)	86 909 (14.52)
2003-2007	3730 (16.47)	3218 (16.25)	15 901 (15.98)	67 300 (17.33)	155 326 (12.96)	74 245 (12.40)
2008-2012	3428 (15.14)	2840 (14.34)	14 030 (14.10)	60 900 (15.68)	149 989 (12.51)	74 306 (12.41)
2013-2016	2336 (10.31)	2035 (10.28)	10 229 (10.28)	43 518 (11.20)	115 054 (9.60)	58 049 (9.70)
Sex						
Male	12 432 (54.89)	10 860 (54.85)	54 012 (54.29)	203 541 (52.40)	608 324 (50.75)	305 756 (51.07)
Female	10 215 (45.11)	8941 (45.15)	45 476 (45.71)	184 875 (47.60)	590 281 (49.25)	292 926 (48.93)
Maternal educational level						
Missing	489 (2.16)	441 (2.23)	2096 (2.11)	9395 (2.42)	25 639 (2.14)	11 135 (1.86)
Primary and lower secondary education	6763 (29.86)	5819 (29.39)	28 715 (28.86)	102 616 (26.42)	313 956 (26.19)	145 525 (24.31)
Upper secondary education and academy profession degree	9715 (42.90)	8480 (42.83)	42 911 (43.13)	167 545 (43.14)	515 308 (42.99)	260 647 (43.54)
Bachelor’s degree and above	5680 (25.08)	5061 (25.56)	25 766 (25.90)	108 860 (28.03)	343 702 (28.68)	181 375 (30.30)
Maternal country of origin						
Missing	40 (0.18)	46 (0.23)	167 (0.17)	572 (0.15)	2126 (0.18)	925 (0.15)
Any country other than Denmark	2682 (11.84)	2135 (10.78)	11 478 (11.54)	52 687 (13.56)	137 990 (11.51)	58 779 (9.82)
Immigrants, descendants, people of Danish origin	19 925 (87.98)	17 620 (88.99)	87 843 (88.30)	335 157 (86.29)	1 058 489 (88.31)	538 978 (90.03)
Parental mental illness						
No history	21 518 (95.01)	18 869 (95.29)	94 837 (95.33)	370 042 (95.27)	1 157 291 (96.55)	580 353 (96.94)
With history	1129 (4.99)	932 (4.71)	4651 (4.67)	18 374 (4.73)	41 314 (3.45)	18 329 (3.06)
Maternal age at time of delivery, y						
12-19	636 (2.81)	556 (2.81)	2666 (2.68)	8573 (2.21)	27 758 (2.32)	12 939 (2.16)
20-24	3739 (16.51)	3245 (16.39)	17 043 (17.13)	61 090 (15.73)	213 663 (17.83)	104 174 (17.40)
25-29	7559 (33.38)	6597 (33.32)	34 623 (34.80)	130 220 (33.53)	443 421 (36.99)	224 181 (37.45)
30-34	6744 (29.78)	6034 (30.47)	29 257 (29.41)	122 147 (31.45)	357 907 (29.86)	181 355 (30.29)
35-39	3268 (14.43)	2829 (14.29)	13 252 (13.32)	55 692 (14.34)	133 838 (11.17)	66 004 (11.02)
≥40	701 (3.10)	540 (2.73)	2647 (2.66)	10 694 (2.75)	22 018 (1.84)	10 029 (1.68)

**Table 2.** Descriptive Statistics of Neuropsychiatric Disorders

Diagnostic categories	*ICD-10* codes	Age at onset, median (IQR), y	Female, No. (%)	Incidence rate, cases/million person-years, No.	Prevalence, cases/1000 births, No.
9 Major types					
Any psychiatric diagnosis	F00-F99	17.0 (11.7-22.1)	1 171 633 (50.3)	5483.3	99.8
Mental and behavioral disorders due to psychoactive substance abuse	F10-F19	21.6 (19.0-25.5)	739 738 (31.8)	577.8	10.9
Schizophrenia related disorders	F20-F29	20.9 (17.9-24.4)	1 056 144 (45.4)	497.7	9.4
Mood disorders	F30-F39	21.5 (17.9-25.9)	1 537 442 (66.1)	1273.0	24.0
Neurotic, stress-related, and somatoform disorders	F40-F48	19.4 (15.6-24.2)	1 423 307 (61.1)	2514.5	46.9
Eating disorders	F50	18.1 (15.4-21.9)	2 175 828 (93.5)	389.7	7.4
Specific personality disorders	F60	21.6 (18.8-25.3)	1 671 078 (71.8)	745.5	14.1
Intellectual disability	F70-F79	12.7 (7.1-17.6)	833 791 (35.8)	313.6	5.9
Pervasive developmental disorders	F84	10.9 (6.7-15.2)	608 291 (26.1)	743.4	14.0
Behavioral and emotional disorders	F90-F98	11.9 (8.3-16.5)	808 301 (34.7)	1761.5	33.0
8 Subtypes of interest					
Mental and behavioral disorders due to alcohol use	F10	24.0 (20.6-28.2)	756 410 (32.5)	182.5	3.5
Mental and behavioral disorders due to cannabis use	F19	21.6 (19.1-25.4)	672 095 (28.9)	199.9	3.8
Schizophrenia	F20	21.6 (19.1-24.9)	1 015 000 (43.6)	247.8	4.7
Schizoaffective disorders	F25	22.5 (19.1-26.8)	1 463 087 (62.9)	21.4	0.4
Bipolar disorder	F30, F31	24.6 (20.8-29.0)	1 439 007 (61.8)	130.0	2.5
Anorexia nervosa	F50.0	16.7 (14.5-20.1)	2 184 965 (93.9)	124.5	2.4
Childhood autism	F84.0	7.5 (4.7-12.4)	486 046 (20.9)	212.2	4.0
Hyperkinetic disorder	F90	12.8 (8.5-19.3)	712 809 (30.6)	1050.4	19.8

Abbreviations: *ICD-10*, *International Statistical Classification of Diseases and Related Health Problems, Tenth Revision*; IQR, interquartile range.

**Figure 1.**
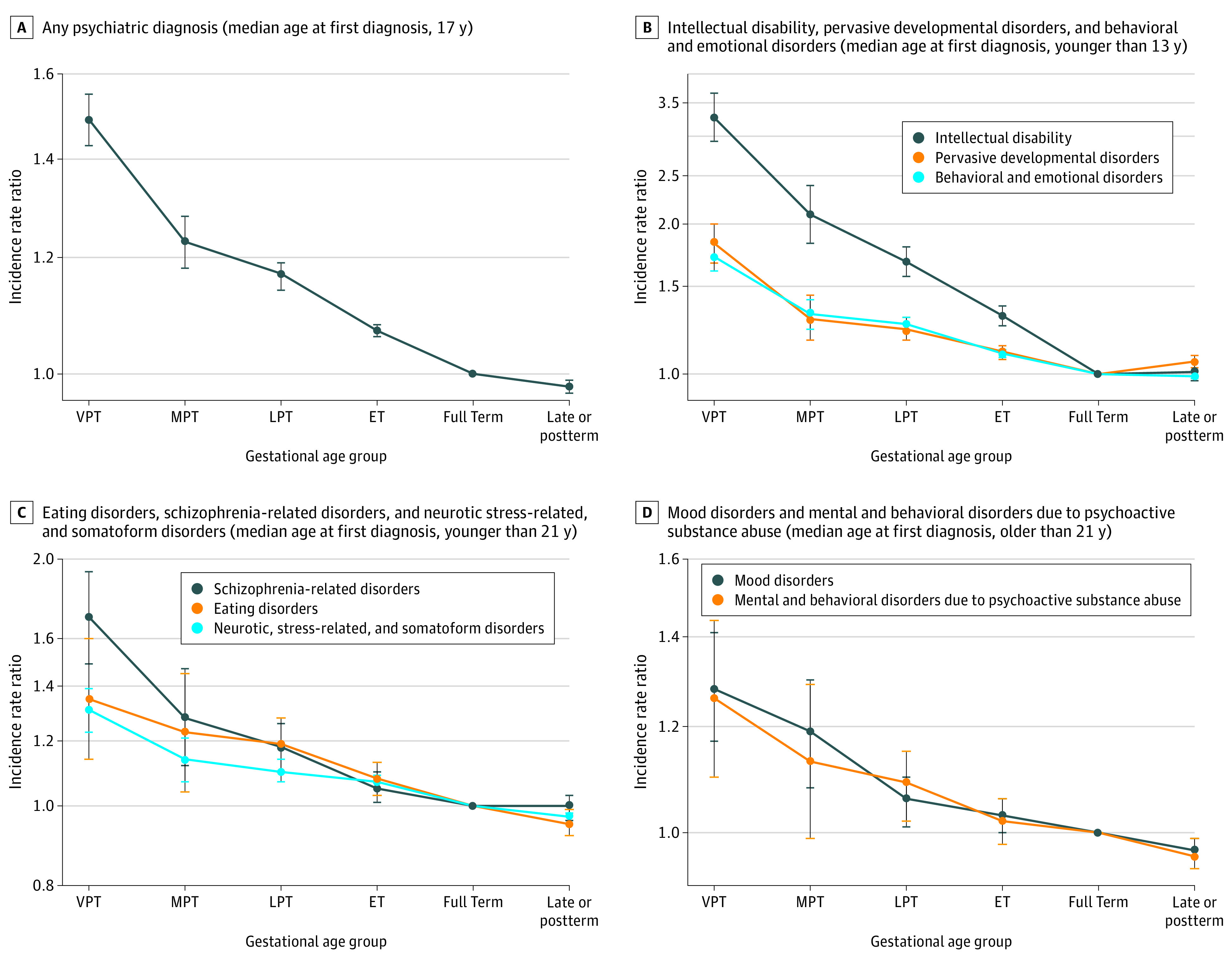
Estimated Incidence Rate Ratios (IRRs) and 95% CIs for Any and 9 Major Neuropsychiatric Disorders by Gestational Age Subgroups Graphs display the associations between the incidence rate of any and each of the 9 major neuropsychiatric disorders and gestational age subgroups: very preterm (VPT; 20-31 weeks), moderate preterm (MPT; 32-33 weeks), late preterm (LPT; 34-36 weeks), early term (ET; 37-38 weeks), full term (reference, 39-40 weeks), and late or postterm (41-45 weeks). Plotted values are IRRs on log scale, and the error bars show 95% CIs. The IRRs were adjusted for sex, calendar year of birth, maternal age at delivery, maternal country of origin, maternal education level, and parental history of mental illness. The crude and the adjusted IRR for all 9 major neuropsychiatric disorders included in the study can be found in eTable 1 in the Supplement.

Figure 1 shows the adjusted IRRs and 95% CIs for any and 9 major neuropsychiatric diagnoses by the 6 gestational age groups. A gradient of decreasing rates with increasing gestational age from very preterm to late preterm was observed for any neuropsychiatric diagnosis compared with term (very preterm: IRR, 1.49 [95% CI, 1.43-1.55]; moderately preterm: IRR, 1.23 [95% CI, 1.18-1.28]; late preterm: IRR, 1.17 [95% CI, 1.14-1.19]). Compared with term births, individuals born early term (37-38 completed weeks) also had a slightly higher incidence rate for any (IRR, 1.07 [95% CI, 1.06-1.08]) and most types of neuropsychiatric diagnoses, whereas a 31% higher rate was estimated for intellectual disability (IRR, 1.31 [95% CI, 1.25-1.37]). Individuals born late or postterm (41 to 45 weeks) had a minor reduction in IRRs for receiving any diagnosis (IRR, 0.98 [95% CI, 0.97-0.99]) compared with term births. The estimated IRRs for the late or postterm group were approximately 2% to 5% lower for diagnosis of mental and behavioral disorders, neurotic and stress-related disorders, eating disorders, and specific personality disorders compared with term, but the IRR was 6% higher for pervasive developmental disorders (IRR, 1.06 [95% CI, 1.03-1.09]). The crude and adjusted IRRs can also be found in eTable 1 in the Supplement.

**Figure 2.**
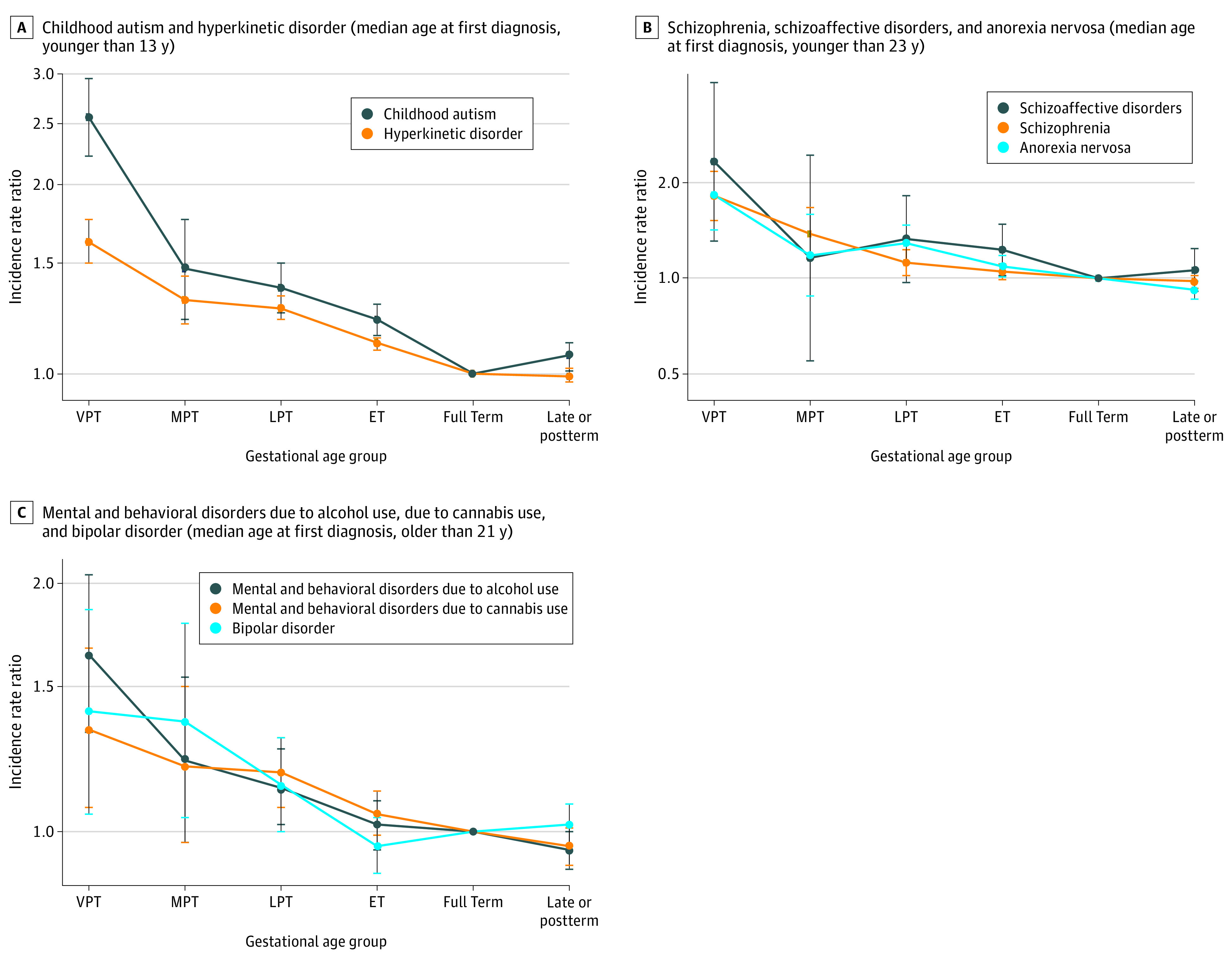
Estimated Incidence Rate Ratios (IRRs) and 95% CIs for 8 Subtypes of Neuropsychiatric Disorders by Gestational Age Subgroups Graphs display the associations between the rate of 8 subtypes of neuropsychiatric disorders and gestational age subgroups: very preterm (VPT; 20-31 weeks), moderate preterm (MPT; 32-33 weeks), late preterm (LPT; 34-36 weeks), early term (ET; 37-38 weeks), full term (reference, 39-40 weeks), and late or postterm (41-45 weeks). Plotted values are IRRs on a log scale, and error bars show 95% CIs. The IRRs were adjusted for sex, calendar year of birth, maternal age at delivery, maternal country of origin, maternal education level, and parental history of mental illness. The crude and the adjusted IRRs for all 8 subtypes included in the study can be found in eTable 2 in the Supplement.

Similar patterns were observed for the 8 subtypes of common neuropsychiatric disorders (Figure 2 and eTable 2 in the Supplement). The IRRs were the highest for all disorders among individuals born very preterm and decreased with increasing gestational age. Individuals born early term had approximately 22% to 23% elevated IRRs for receiving diagnoses of childhood autism (IRR, 1.22 [95% CI, 1.15-1.29]) and schizoaffective disorders (IRR, 1.23 [95% CI, 1.02-1.48]), compared with individuals born at term. Only the estimated IRR for childhood autism was elevated (7% higher) among individuals born postterm when compared with term (IRR, 1.07 [95% CI, 1.01-1.12]).

Among 224 735 individuals who ever received a diagnosis for any major neuropsychiatric disorders, 54.8% had 1 diagnosis, 39.0% had 2 to 3 diagnoses, and 6.2% had 4 or more types of neuropsychiatric disorders. A shorter gestational age at birth was associated with an increasing number of psychiatric comorbidities (Table 3 and eFigure in the Supplement). For instance, very preterm births (20-31 weeks) had 15% higher odds of receiving a diagnosis for only 1 type of neuropsychiatric disorder (OR, 1.15 [95% CI, 1.08-1.22]), 41% higher odds of receiving 2 or 3 diagnoses (OR, 1.41 [95% CI, 1.32-1.51]), and 50% higher odds of receiving 4 or more diagnoses (OR, 1.50 [95% CI, 1.28-1.77]) compared with term births (39-40 weeks).

**Table 3.** Adjusted ORs and 95% CIs for the Number of Comorbid Major Neuropsychiatric Disorders by Gestational Age Subgroups^a^

Gestational age subgroup	Individuals, No.	Only 1 neuropsychiatric diagnosis		2-3 Neuropsychiatric diagnoses		≥4 Neuropsychiatric diagnoses	
		Individuals, No. (%)	OR (95% CI)	Individuals, No. (%)	OR (95% CI)	Individuals, No. (%)	OR (95% CI)
Very preterm (20-31 wk)	22 647	1180 (50.6)	1.15 (1.08-1.22)	995 (42.7)	1.41 (1.32-1.51)	155 (6.7)	1.50 (1.28-1.76)
Moderate preterm (32-33 wk)	19 801	1128 (53.1)	1.14 (1.07-1.21)	836 (39.3)	1.21 (1.13-1.30)	161 (7.6)	1.57 (1.34-1.84)
Late preterm (34-36 wk)	99 488	5788 (54.5)	1.15 (1.11-1.18)	4168 (39.3)	1.18 (1.14-1.22)	660 (6.2)	1.23 (1.14-1.34)
Early term (37-38 wk)	388 416	20 041 (55.0)	1.06 (1.04-1.07)	14 293 (39.2)	1.09 (1.07-1.11)	2132 (5.8)	1.10 (1.05-1.16)
Term (39-40 wk)	1 198 605	63 901 (54.9)	1.00 [Reference]	45 283 (38.9)	1.00 [Reference]	7262 (6.2)	1.00 [Reference]
Postterm (41-45 wk)	598 682	31 165 (54.9)	0.98 (0.97-0.99)	22 135 (39.0)	0.99 (0.97-1.00)	3452 (6.1)	0.97 (0.93-1.01)

^a^ Odds ratios (ORs) and 95% CIs were estimated using multinomial logistic regression model with no diagnosis of any neuropsychiatric disorders as the reference for the 4-level outcome. Model was adjusted for sex, calendar year of birth, maternal age at delivery, maternal place of origin, maternal education level, and parental history of mental illness.

In a sensitivity analysis, the overall findings remained largely unchanged when the study population only included individuals born since January 1, 1995, with slight decreases in some effect sizes for disorders with later age of onset requiring a longer follow-up (eTable 3 in the Supplement). In sex-stratified analyses, only small differences were found for any neuropsychiatric diagnosis where the IRRs were higher for very preterm and late preterm male individuals, and the IRR between a shorter gestational duration and schizophrenia was higher for female individuals (eTable 4 in the Supplement).

## Discussion

This study provides a detailed assessment of the association between finer categorizations of gestational age groups and the incidence rates for major childhood and adult neuropsychiatric disorders in Denmark. We found that diagnosis of both child-onset and adult-onset neuropsychiatric diseases was associated with preterm birth, with a gradient of risk observed from very preterm (20-31 weeks) to late preterm (34-36 weeks) birth. Our study also provides evidence of heterogeneity in long-term neuropsychiatric risk by gestational age within the term spectrum. Early term births (37-38 weeks), usually considered as a low-risk group in previous studies, had a slightly elevated rate for multiple neuropsychiatric disorders. Late or postterm births (41-45 weeks) had the lowest rates for most of the major and subtypes of neuropsychiatric disorders evaluated, except pervasive developmental disorders, specifically childhood autism. In addition, our data illustrated that gestational age at birth was associated with comorbidity of major neuropsychiatric disorders, suggesting possible shared mechanisms for disease cause or susceptibility stemming from impaired brain development early in life.

Most previous studies^4,5,7,9^ primarily focused on diagnosis in childhood according to 2 (term or preterm) or 3 (term, preterm, and very preterm) levels of classification regarding gestational age at birth. A few recent studies^18,28,29,30^ have evaluated the heterogeneity of long-term neuropsychiatric risk among individuals born beyond 37 gestational weeks, reporting findings consistent with our results showing slightly elevated rates of neuropsychiatric disorders among the early term births. A recent study^14^ has demonstrated that early term births born at 37 to 39 weeks might have poorer educational outcomes compared with those born at 39 to 41 weeks of gestation. There has also been evidence on postterm delivery and mental health disorders.^17,18,31^ However, unlike the Helsinki Birth Cohort Study,^17,31^ we did not find elevated risks for other disorders associated with prolonged gestation except pervasive development disorders, specifically childhood autism.

Mechanisms linking gestational age with later risk of neuropsychiatric disorders are likely to be multifactorial.^3^ With regard to preterm birth, placental insufficiency might be associated with both the development of the brain and preterm or early term birth because of fetal growth restriction or preeclampsia.^32^ The last trimester of pregnancy is a critical period for brain development and growth, when multiple important neurobiological processes are actively taking place, including substantial increases in cerebral volume,^33^ rapid gyrification,^34^ and formation of cortical connections.^35^ Insufficient development in late gestation could affect extensive structural brain alterations^36,37^ and/or alterations in other developmentally regulated processes^38^ that underlie long-term neuropsychiatric deficits. Prematurity might also act as a surrogate for the effects of adverse obstetric circumstances or environmental and social stressors^39,40,41,42,43^ on neurodevelopment and mental health.^38,44,45^ Moreover, infants born prematurely might be exposed to neurotoxic chemicals^46^ and medications^47^ when admitted to the neonatal intensive care unit. We have controlled for several related maternal sociodemographic factors and parental mental illnesses in the analyses; however, confounding by a wider range of risk factors, including genetic factors, remains possible.^48^ The observed sex differences in some associations might be due to different profiles of obstetric insults and brain anomalies in male and female individuals, especially among individuals with schizophrenia,^49^ but they need to be further evaluated.

Among the traditionally perceived homogeneous low-risk gestational age groups, individuals born early term might also be subject to physiological^13,50^ and brain^50^ insults and alterations after birth, because the brain is only 90% of full-term weight even at 38 weeks of gestation.^51^ The association between late or postterm birth and elevated risk for pervasive developmental disorders, including childhood autism, requires further investigations of unique risk factors that might be associated with increased risk of these disorders. For instance, recent research concerning the effects of approaches for managing prolonged pregnancy has observed a slightly elevated risk for autism among male individuals in the Danish birth cohort associated with oxytocin-augmented labor,^52^ yet such the associations have not been confirmed in a Swedish population study using a sibling-matched design.^53^

### Strengths and Limitations

Our study has several strengths. First, a long-term follow-up of a nationwide population-based sample allowed us to quantify incidence rates for multiple neuropsychiatric diagnoses. The large sample size enabled us to compare gestational age at birth categorized into 6 groups according to current clinical and research practices. Furthermore, information on gestational age and parental sociodemographic factors were collected prospectively and recorded independently of the outcome diagnoses.^19,21^ Neuropsychiatric diagnoses were ascertained by trained psychiatrists on the basis of standardized *ICD-10* diagnostic criteria recorded in the DPCR.^54^ Systematic studies have not been conducted to evaluate the validity of records for all outcomes, but validations for some specific disorders^22,55,56^ have been performed, suggesting good validity for research.

This study also has some limitations. First, misclassification of the exposure is present to some degree. A previous Danish study^57^ compared gestational age between medical records and registry data and found a 2-week difference (plus or minus) among approximately 90% of those with disagreement. Although the classification for more extreme values (eg, very preterm birth) is expected to have a higher validity,^58^ classifications of other gestational subgroups might be subject to misclassification. For instance, it is possible that individuals born late preterm were misclassified as early term, contributing to the observed small increase in risk for early term births, whereas a misclassification in the opposite direction can also occur, leading to diluted effect sizes.^19^ Second, patients treated by private practicing psychiatrists in Denmark were not reported to the DPCR and, therefore, were not captured in our study. However, the associations of such underdetection with outcomes are expected to be minimal in Denmark.^54^ Third, diagnoses during 1978 to 1994 made in *ICD-8* codes were omitted, leading to possible underestimation of disease rates, particularly for the disorders with an early onset. Our results remained largely unchanged in analyses restricted to individuals born after 1994, when *ICD-10* codes were used in Denmark, suggesting that the effect of excluding *ICD-8* diagnoses was minimal. Furthermore, other subtypes of neuropsychiatric disorders and fetal development indicators, including intrauterine growth restriction, that were not examined here warrant further investigation. Fourth, other perinatal risk factors, such as maternal lifestyle, pregnancy complications, and environmental exposures during the prenatal and postnatal periods,^59,60^ have likely contributed to the observed associations. Future research that assesses the role of these factors for long-term neuropsychiatric risk could provide mechanistic insights and inform intervention strategies.

## Conclusions

This cohort study provides a comprehensive assessment of incidence rates for major neuropsychiatric disorders according to finer classifications of gestational age at birth in Denmark. Gestational age, not only across the spectrum of preterm but also beyond the conventional threshold of term, is associated with occurrences of single and multiple neuropsychiatric disorders later in life. Our findings suggest that childhood and adult neuropsychiatric disorders might stem from factors related to early development. Intervention strategies targeted at perinatal risk factors and obstetric practices preventing nonoptimal delivery timing and improving postnatal care for those born with nonoptimal gestational duration might reduce long-term neuropsychiatric risk in the population.

## Notes

Supplement. **eFigure.** Adjusted Odds Ratios and 95% Confidence Intervals for the Number of Comorbid Major Neuropsychiatric Disorders by Gestational Age Subgroups **eTable 1.** Estimated Incidence Rate Ratio and 95% Confidence Intervals for Any and Each of the Nine Major Neuropsychiatric Disorders by Gestational Age Subgroups **eTable 2.** Estimated Incidence Rate Ratios and 95% Confidence Intervals (CI) for Each of the Eight Subtypes of Neuropsychiatric Disorders by Gestational Age Subgroups **eTable 3.** Estimated Incidence Rate Ratios and 95% Confidence Intervals for Neuropsychiatric Disorders by Gestational Age Subcategories, Restricted to Individuals Born 1995 and Onward **eTable 4.** Estimated Incidence Rate Ratios and 95% Confidence Intervals (CI) for Any and Each of the Nine Major Neuropsychiatric Disorders by Gestational Age Subgroups, Stratified by Sex
