# Supplementary material for: Rates of Neuropsychiatric Disorders and Gestational Age at Birth in a Danish Population
Source: JAMA Netw Open. 2021 Jun 29;4(6):e2114913. doi: 10.1001/jamanetworkopen.2021.14913 (PMC8243234; doi:10.1001/jamanetworkopen.2021.14913)
Supplement: Supplement. — eFigure. Adjusted Odds Ratios and 95% Confidence Intervals for the Number of Comorbid Major Neuropsychiatric Disorders by Gestational Age Subgroups eTable 1. Estimated Incidence Rate Ratio and 95% Confidence Intervals for Any and Each of the Nine Major Neuropsychiatric Disorders by Gestational Age Subgroups eTable 2. Estimated Incidence Rate Ratios and 95% Confidence Intervals (CI) for Each of the Eight Subtypes of Neuropsychiatric Disorders by Gestational Age Subgroups eTable 3. Estimated Incidence Rate Ratios and 95% Confidence Intervals for Neuropsychiatric Disorders by Gestational Age Subcategories, Restricted to Individuals Born 1995 and Onward eTable 4. Estimated Incidence Rate Ratios and 95% Confidence Intervals (CI) for Any and Each of the Nine Major Neuropsychiatric Disorders by Gestational Age Subgroups, Stratified by Sex [file jamanetwopen-e2114913-s001.pdf]

Supplemental Online Content

Xia Y, Xiao J, Yu Y, et al. Rates of neuropsychiatric disorders and gestational age at birth in a Danish population. *JAMA Netw Open*. 2021;4(6):e2114913. doi:10.1001/jamanetworkopen.2021.14913

eFigure. Adjusted Odds Ratios and 95% Confidence Intervals for the Number of Comorbid Major Neuropsychiatric Disorders by Gestational Age Subgroups
eTable 1. Estimated Incidence Rate Ratio and 95% Confidence Intervals for Any and Each of the Nine Major Neuropsychiatric Disorders by Gestational Age Subgroups
eTable 2. Estimated Incidence Rate Ratios and 95% Confidence Intervals (CI) for Each of the Eight Subtypes of Neuropsychiatric Disorders by Gestational Age Subgroups
eTable 3. Estimated Incidence Rate Ratios and 95% Confidence Intervals for Neuropsychiatric Disorders by Gestational Age Subcategories, Restricted to Individuals Born 1995 and Onward
eTable 4. Estimated Incidence Rate Ratios and 95% Confidence Intervals (CI) for Any and Each of the Nine Major Neuropsychiatric Disorders by Gestational Age Subgroups, Stratified by Sex

This supplemental material has been provided by the authors to give readers additional information about their work.

© 2021 Xia Y et al. *JAMA Network Open.*

eFigure. Adjusted Odds Ratios and 95% Confidence Intervals for the Number of Comorbid Major Neuropsychiatric Disorders by Gestational Age Subgroups

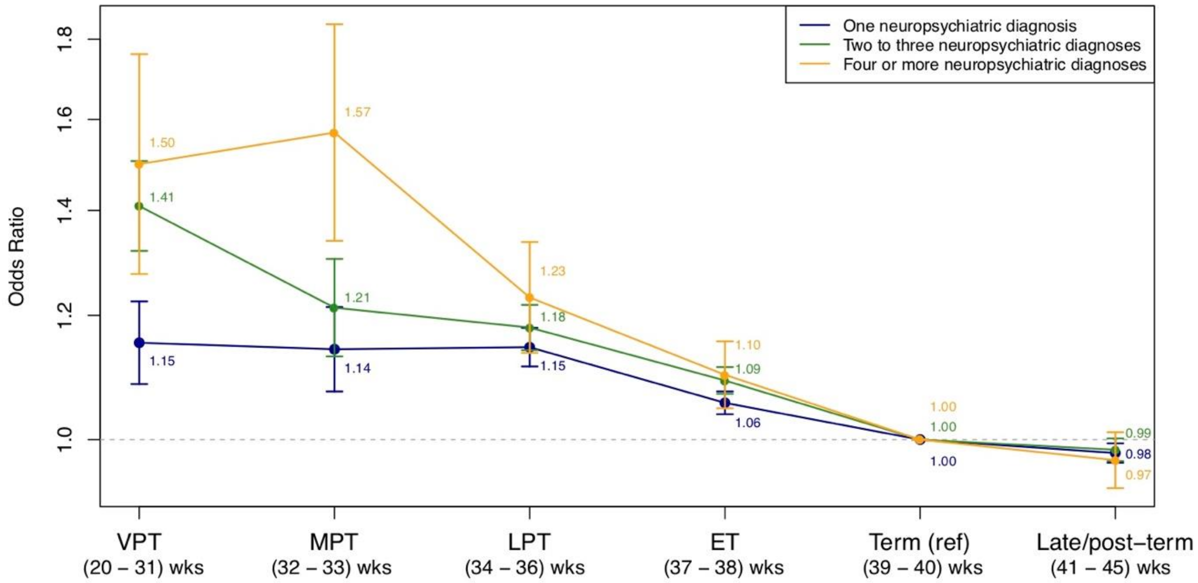

eFigure displays the estimated associations between the number of comorbid neuropsychiatric conditions and gestational age subgroups (very preterm (VPT), moderate preterm (MPT), late preterm (LPT), early term (ET), term [reference], and late/post-term). Plotted values are odds ratios (IRR) on log scale, and the tick marks show 95% confidence intervals (CI). Values were adjusted for sex, calendar year of birth, maternal age at delivery, maternal country of origin, maternal education level, and parental history of mental illness. The blue line represents having received one neuropsychiatry diagnosis, the green line represents having two to three diagnoses, and the yellow line presents having received four or more neuropsychiatric diagnoses recorded in the Danish Psychiatric Central Registry.

© 2021 Xia Y et al. *JAMA Network Open.*

eTable 1. Estimated Incidence Rate Ratio and 95% Confidence Intervals for Any and Each of the Nine Major Neuropsychiatric Disorders by Gestational Age Subgroups

	Incidence Rate Ratios (IRRs) and 95% CI [a]											
	Very preterm		Moderate preterm		Late preterm		Early term		Term		Post term	
	(20 - 31) weeks		(32 - 33) weeks		(34 - 36) weeks		(37 - 38) weeks		(39 - 40) weeks		(41 - 45) weeks	
	Crude	Adjusted	Crude	Adjusted	Crude	Adjusted	Crude	Adjusted	Crude	Adjusted	Crude	Adjusted
Any Psychiatric Diagnosis	1.58	1.49	1.29	1.23	1.22	1.17	1.10	1.07	Ref	Ref	0.99	0.98
	(1.57, 1.59)	(1.43, 1.55)	(1.28, 1.30)	(1.18, 1.28)	(1.21, 1.22)	(1.14, 1.19)	(1.10, 1.10)	(1.06, 1.08)			(0.99, 0.99)	(0.97, 0.99)
Mental and Behavioral Disorders Due to Psychoactive Substance Abuse	1.20	1.26	1.12	1.13	1.08	1.09	0.95	1.02	Ref	Ref	0.94	0.96
	(1.17, 1.23)	(1.10, 1.44)	(1.09, 1.15)	(0.99, 1.29)	(1.07, 1.10)	(1.02, 1.15)	(0.94, 0.95)	(0.98, 1.06)			(0.94, 0.95)	(0.94, 0.99)
Schizophrenia Related Disorders	1.62	1.70	1.25	1.28	1.16	1.18	1.00	1.05	Ref	Ref	0.99	1.00
	(1.59, 1.66)	(1.49, 1.93)	(1.22, 1.28)	(1.12, 1.47)	(1.15, 1.17)	(1.10, 1.26)	(0.99, 1.00)	(1.01, 1.10)			(0.98, 0.99)	(0.96, 1.03)
Mood Disorders	1.15	1.28	1.09	1.19	0.99	1.06	0.94	1.03	Ref	Ref	0.97	0.97
	(1.13, 1.17)	(1.17, 1.41)	(1.07, 1.11)	(1.08, 1.30)	(0.98, 1.00)	(1.01, 1.10)	(0.93, 0.94)	(1.00, 1.06)			(0.97, 0.97)	(0.96, 0.99)
Neurotic, Stress-related, and Somatoform Disorders	1.28	1.31	1.12	1.14	1.09	1.10	1.03	1.07	Ref	Ref	0.97	0.97
	(1.27, 1.30)	(1.23, 1.39)	(1.11, 1.14)	(1.07, 1.21)	(1.09, 1.10)	(1.07, 1.14)	(1.03, 1.04)	(1.05, 1.09)			(0.96, 0.97)	(0.95, 0.98)
Eating Disorders	1.19	1.35	1.08	1.23	1.07	1.19	0.99	1.08	Ref	Ref	0.97	0.95
	(1.15, 1.22)	(1.14, 1.60)	(1.05, 1.11)	(1.04, 1.45)	(1.05, 1.08)	(1.10, 1.28)	(0.98, 0.99)	(1.03, 1.13)			(0.96, 0.98)	(0.92, 0.99)
Specific Personality Disorders	1.18	1.37	1.16	1.28	1.04	1.13	0.97	1.09	Ref	Ref	0.95	0.97
	(1.16, 1.21)	(1.21, 1.54)	(1.14, 1.18)	(1.14, 1.44)	(1.03, 1.05)	(1.07, 1.19)	(0.96, 0.97)	(1.06, 1.13)			(0.94, 0.95)	(0.94, 0.99)
Intellectual Disability	4.17	3.27	2.55	2.09	2.00	1.68	1.53	1.31	Ref	Ref	1.02	1.01
	(4.09, 4.25)	(2.93, 3.66)	(2.49, 2.61)	(1.83, 2.39)	(1.97, 2.02)	(1.57, 1.80)	(1.52, 1.55)	(1.25, 1.37)			(1.02, 1.03)	(0.97, 1.06)
Pervasive Developme	2.43	1.83	1.63	1.29	1.51	1.23	1.37	1.11	Ref	Ref	1.10	1.06

© 2021 Xia Y et al. *JAMA Network Open.*

ntal Disorders	(2.39, 2.47)	(1.67, 2.00)	(1.60, 1.66)	(1.17, 1.44)	(1.49, 1.52)	(1.17, 1.30)	(1.36, 1.38)	(1.07, 1.14)			(1.09, 1.11)	(1.03, 1.09)
Behavioral and Emotional Disorders	2.17	1.72	1.60	1.32	1.50	1.26	1.30	1.10	Ref	Ref	1.01	0.99
	(2.15, 2.20)	(1.61, 1.83)	(1.62, 1.49)	(1.23, 1.41)	(1.51, 1.29)	(1.22, 1.30)	(1.30, 1.01)	(1.08, 1.12)			(1.02, 1.02)	(0.98, 1.01)

[a]Adjusted for sex, calendar year of birth, maternal age at delivery, maternal country of origin, maternal education level, and parental history of mental illness

© 2021 Xia Y et al. *JAMA Network Open.*

eTable 2. Estimated Incidence Rate Ratios and 95% Confidence Intervals (CI) for Each of the Eight Subtypes of Neuropsychiatric Disorders by Gestational Age Subgroups

	Incidence Rate Ratios (IRRs) and 95% CI [a]											
	Very preterm		Moderate preterm		Late preterm		Early term		Term		Post term	
	(20 - 31) weeks		(32 - 33) weeks		(34 - 36) weeks		(37 - 38) weeks		(39 - 40) weeks		(41 - 45) weeks	
	Crude	Adjusted	Crude	Adjusted	Crude	Adjusted	Crude	Adjusted	Crude	Adjusted	Crude	Adjusted
Mental and Behavioral Disorders Due to Alcohol Use	1.43	1.64	1.15	1.22	1.08	1.13	0.89	1.02	Ref	Ref	0.90	0.95
	(1.38, 1.49)	(1.32, 2.05)	(1.10, 1.20)	(0.97, 1.54)	(1.06, 1.10)	(1.02, 1.26)	(0.88, 0.90)	(0.95, 1.09)			(0.89, 0.91)	(0.90, 1.00)
Mental and Behavioral Disorders Due to Cannabis Use	1.27	1.33	1.21	1.20	1.19	1.18	0.98	1.05	Ref	Ref	0.93	0.96
	(1.22, 1.32)	(1.07, 1.67)	(1.16, 1.25)	(0.97, 1.50)	(1.17, 1.20)	(1.07, 1.30)	(0.97, 0.99)	(0.99, 1.12)			(0.92, 0.93)	(0.91, 1.01)
Schizophrenia	1.67	1.82	1.32	1.38	1.08	1.12	0.96	1.05	Ref	Ref	0.96	0.98
	(1.62, 1.73)	(1.52, 2.17)	(1.27, 1.36)	(1.15, 1.67)	(1.06, 1.10)	(1.02, 1.23)	(0.95, 0.97)	(0.99, 1.11)			(0.95, 0.97)	(0.93, 1.02)
Schizoaffective Disorders	1.91	2.33	1.00	1.16	1.18	1.33	1.05	1.23	Ref	Ref	1.03	1.06
	(1.72, 2.11)	(1.31, 4.13)	(0.88, 1.15)	(0.55, 2.44)	(1.12, 1.25)	(0.97, 1.82)	(1.02, 1.08)	(1.02, 1.48)			(1.00, 1.06)	(0.91, 1.24)
Bipolar Disorder	1.17	1.40	1.20	1.36	1.02	1.14	0.84	0.96	Ref	Ref	1.00	1.02
	(1.11, 1.23)	(1.05, 1.86)	(1.14, 1.25)	(1.04, 1.79)	(1.00, 1.05)	(1.00, 1.30)	(0.83, 0.85)	(0.89, 1.04)			(0.99, 1.01)	(0.96, 1.08)
Anorexia Nervosa	1.63	1.83	1.05	1.18	1.16	1.29	1.01	1.09	Ref	Ref	0.94	0.92
	(1.56, 1.71)	(1.42, 2.35)	(0.99, 1.10)	(0.88, 1.59)	(1.13, 1.19)	(1.13, 1.47)	(1.00, 1.02)	(1.01, 1.18)			(0.93, 0.95)	(0.86, 0.98)
Childhood Autism	3.73	2.56	1.99	1.47	1.78	1.37	1.63	1.22	Ref	Ref	1.11	1.07
	(3.64, 3.83)	(2.22, 2.95)	(1.92, 2.05)	(1.22, 1.76)	(1.75, 1.81)	(1.25, 1.50)	(1.62, 1.65)	(1.15, 1.29)			(1.10, 1.12)	(1.01, 1.12)
Hyperkinetic Disorder	2.08	1.62	1.61	1.31	1.53	1.27	1.32	1.12	Ref	Ref	1.01	0.99
	(2.05, 2.11)	(1.50, 1.76)	(1.58, 1.63)	(1.20, 1.43)	(1.52, 1.54)	(1.22, 1.33)	(1.32, 1.33)	(1.09, 1.14)			(1.01, 1.02)	(0.97, 1.02)

[a] Adjusted for sex, calendar year of birth, maternal age at delivery, maternal country of origin, maternal education level, and parental history of mental illness)

© 2021 Xia Y et al. *JAMA Network Open.*

eTable 3. Estimated Incidence Rate Ratios and 95% Confidence Intervals for Neuropsychiatric Disorders by Gestational Age Subcategories, Restricted to Individuals Born 1995 and Onward

Diagnostic Categories	Incidence Rate Ratios (IRRs) and 95% CI[a]					
	Very preterm	Moderate preterm	Late preterm	Early term	Term	Post term
	(20 - 31) weeks	(32 - 33) weeks	(34 - 36) weeks	(37 - 38) weeks	(39 - 40) weeks	(41 - 45) weeks
Any Psychiatric Diagnosis	1.58	1.24	1.21	1.08	Ref	1.00
	(1.49, 1.67)	(1.16, 1.32)	(1.17, 1.24)	(1.06, 1.10)		(0.98, 1.02)
Mental and Behavioral Disorders Due to Psychoactive Substance Abuse	1.13	0.94	1.08	0.97	Ref	0.97
	(0.80, 1.59)	(0.64, 1.37)	(0.92, 1.27)	(0.88, 1.07)		(0.89, 1.06)
Schizophrenia Related Disorders	1.41	1.14	1.16	1.06	Ref	1.03
	(1.07, 1.85)	(0.84, 1.54)	(1.01, 1.33)	(0.97, 1.15)		(0.96, 1.11)
Mood Disorders	1.12	0.99	1.02	0.99	Ref	0.96
	(0.92, 1.36)	(0.80, 1.21)	(0.93, 1.12)	(0.93, 1.04)		(0.92, 1.01)
Neurotic, Stress-related, and Somatoform Disorders	1.24	1.08	1.11	1.06	Ref	0.98
	(1.12, 1.38)	(0.97, 1.21)	(1.05, 1.17)	(1.03, 1.10)		(0.95, 1.01)
Eating Disorders	1.55	1.06	1.23	1.12	Ref	0.94
	(1.20, 2.00)	(0.79, 1.44)	(1.08, 1.39)	(1.04, 1.21)		(0.88, 1.01)
Specific Personality Disorders	1.33	1.13	1.09	1.12	Ref	1.04
	(0.98, 1.81)	(0.81, 1.58)	(0.94, 1.27)	(1.02, 1.22)		(0.96, 1.12)
Intellectual Disability	2.75	1.88	1.63	1.26	Ref	1.02
	(2.37, 3.19)	(1.57, 2.24)	(1.49, 1.79)	(1.19, 1.33)		(0.96, 1.08)
Pervasive Developmental Disorders	1.74	1.22	1.21	1.09	Ref	1.06
	(1.57, 1.93)	(1.08, 1.38)	(1.15, 1.29)	(1.06, 1.13)		(1.03, 1.09)
Behavioral and Emotional Disorders	1.76	1.32	1.29	1.11	Ref	1.00
	(1.63, 1.89)	(1.21, 1.43)	(1.24, 1.34)	(1.09, 1.14)		(0.98, 1.03)

[a] Adjusted for sex, calendar year of birth, maternal age at delivery, maternal country of origin, maternal education level, and parental history of mental illness)

© 2021 Xia Y et al. *JAMA Network Open.*

eTable 4. Estimated Incidence Rate Ratios and 95% Confidence Intervals (CI) for Any and Each of the Nine Major Neuropsychiatric Disorders by Gestational Age Subgroups, Stratified by Sex

Diagnostic Categories		Incidence Rate Ratios (IRRs) and 95% CI for Diagnoses by Gestational Age Stage					
		Very preterm	Moderate preterm	Late preterm	Early term	Term	Post term
		(20 - 31) weeks	(32 - 33) weeks	(34 - 36) weeks	(37 - 38) weeks	(39 - 40) weeks	(41 - 45) weeks
Any Psychiatric Diagnosis	Male	1.57	1.23	1.19	1.06	Ref	0.99
		(1.48, 1.65)	(1.16, 1.30)	(1.16, 1.22)	(1.05, 1.08)		(0.98, 1.00)
	Female	1.40	1.26	1.14	1.08	Ref	0.97
		(1.32, 1.49)	(1.14, 1.30)	(1.11, 1.17)	(1.06, 1.10)		(0.96, 0.99)
	P-value	<0.01	0.09	<0.01	<0.01	Ref	0.05
Mental and Behavioral Disorders Due to Psychoactive Substance Abuse	Male	1.23	1.09	1.07	1.01	Ref	0.98
		(1.05, 1.45)	(0.93, 1.28)	(1.00, 1.16)	(0.96, 1.05)		(0.95, 1.02)
	Female	1.33	1.23	1.12	1.04	Ref	0.92
		(1.04, 1.70)	(0.97, 1.56)	(1.00, 1.25)	(0.98, 1.11)		(0.88, 0.97)
	P-value	0.40	0.30	0.33	0.09	Ref	0.08
Schizophrenia Related Disorders	Male	1.44	1.21	1.09	0.99	Ref	0.98
		(1.20, 1.73)	(1.01, 1.46)	(1.00, 1.19)	(0.94, 1.04)		(0.94, 1.02)
	Female	2.06	1.38	1.29	1.14	Ref	1.02
		(1.72, 2.46)	(1.12, 1.69)	(1.17, 1.42)	(1.08, 1.21)		(0.97, 1.07)
	P-value	<0.01	0.24	<0.01	<0.01	Ref	0.07
Mood Disorders	Male	1.25	1.10	1.05	0.96	Ref	0.98
		(1.07, 1.46)	(0.94, 1.28)	(0.98, 1.13)	(0.92, 1.01)		(0.95, 1.01)
	Female	1.30	1.24	1.06	1.07	Ref	0.97
		(1.16, 1.46)	(1.11, 1.39)	(1.03, 1.10)	(1.03, 1.10)		(0.95, 1.00)
	P-value	0.59	0.20	0.78	<0.01	Ref	0.91
Neurotic, Stress-related, and Somatoform Disorders	Male	1.30	1.06	1.08	1.05	Ref	0.97
		(1.18, 1.54)	(0.95, 1.17)	(1.3, 1.13)	(1.02, 1.08)		(0.94, 0.99)
	Female	1.31	1.20	1.12	1.08	Ref	0.97
		(1.21, 1.43)	(1.10, 1.30)	(1.07, 1.17)	(1.05, 1.10)		(0.95, 0.98)
	P-value	0.99	0.08	0.44	0.46	Ref	0.81
Eating Disorders	Male	2.15	0.98	1.25	1.09	Ref	0.95
		(1.34, 3.43)	(0.51, 1.90)	(0.96, 1.63)	(0.93, 1.29)		(0.82, 1.10)
	Female	1.28	1.25	1.18	1.08	Ref	0.95
		(1.07, 1.53)	(1.05, 1.48)	(1.09, 1.28)	(1.03, 1.13)		(0.91, 0.99)
	P-value	0.01	0.68	0.34	0.22	Ref	0.78
Specific Personality Disorders	Male	1.37	1.21	1.18	1.07	Ref	0.97
		(1.10, 1.70)	(0.97, 1.49)	(1.07, 1.30)	(1.00, 1.13)		(0.92, 1.02)
	Female	1.37	1.32	1.11	1.10	Ref	0.97
		(1.19, 1.58)	(1.15, 1.52)	(1.04, 1.19)	(1.06, 1.15)		(0.94, 1.00)
	P-value	0.65	0.34	0.63	0.04	Ref	0.58
Intellectual Disability	Male	3.13	2.05	1.57	1.25	Ref	1.03
		(2.73, 3.59)	(1.74, 2.41)	(1.44, 1.71)	(1.18, 1.32)		(0.98, 1.09)
	Female	3.57	2.18	1.90	1.44	Ref	0.97
		(2.96, 4.3)	(1.73, 2.74)	(1.69, 2.13)	(1.33, 1.55)		(0.90, 1.04)
	P-value	0.58	0.93	0.04	0.10	Ref	0.12
Pervasive Developmental Disorders	Male	1.67	1.29	1.22	1.10	Ref	1.00
		(1.50, 1.86)	(1.13, 1.46)	(1.15, 1.30)	(1.07, 1.14)		(0.97, 1.03)
	Female	1.51	1.17	1.11	1.01	Ref	0.95
		(1.15, 1.98)	(0.88, 1.56)	(0.97, 1.26)	(0.93, 1.09)		(0.89, 1.02)
	P-value	0.00	0.36	0.02	0.11	Ref	0.75
Behavioral and Emotional Disorders	Male	1.97	1.34	1.20	1.04	Ref	0.94
		(1.36, 2.85)	(0.89, 2.02)	(0.99, 1.46)	(0.92, 1.17)		(0.85, 1.03)
	Female	1.31	1.09	1.15	1.03	Ref	0.99
		(1.00, 1.71)	(0.83, 1.43)	(1.02, 1.29)	(0.96, 1.11)		(0.93, 1.05)
	P-value	0.32	0.46	0.07	0.13	Ref	0.34

[a] Adjusted for calendar year of birth, maternal age at delivery, maternal country of origin, maternal education level, and parental history of mental illness. P-value for heterogeneity was assessed by including a sex and gestational age group product term in the model.

© 2021 Xia Y et al. *JAMA Network Open.*
